# Case of Recovery of a Patient after Swallowing a Large Quantity of Sulphuric Acid

**Published:** 1818-07

**Authors:** J. D. Browne

**Affiliations:** Member of the Royal College of Surgeons, London.


*For the London Medical and Physical Journal.*

*Case of Recovery of a Patient after swallowing a large quantity of Sulphuric Acid;* by J. D. Browne, Member of the Royal College of Surgeons, London.

IN the afternoon of the 17th of March, 1818, I was called to a Mrs. ——, in this neighbourhood, who was supposed to be dying from the effects of a quantity of vitriolic acid, which she had taken with an intention of destroying life. I visited her about half an hour after her vomiting it, and found her in a most distressing state, labouring under severe pain of the stomach and bowels: pulse imperceptible; extremities cold; insensible; with a slight degree of vomiting, evidently of a powerful acid, it destroying every thing coming in contact with it, and, where some had been ejected on the floor, the boards were evidently darkened. I immediately gave her pulv. emet. com.; and, after emptying the contents of the stomach, endeavoured to throw in the potassæ subcarb. and magnesia, but to no effect,—the stomach rejecting every thing instantly when administered.

18th.—The vomiting still continuing, impregnated with a strong acid,—complained of great soreness of the fauces, œsophagus, &c. with violent pain of the stomach and bowels; extremities still cold, and great irregularity of pulse, with occasional expectoration of blood: examined the mouth, perceived several excoriations, but of no particular consequence: continued the magnesia at intervals, but unsuccessfully; applied emp. lyttæ over the epigastric region, and ordered an enema. Visited in the evening: vomiting had continued incessantly during the day, but the contents discharged had altered in appearance evidently, being the mere common secretion of the stomach slightly acidulated. Repeated the enema.

19th.—Vomiting had somewhat abated, having retained the magnesia in small doses. Pain of the stomach and bowels rather alleviated; circulation in the extremities increased: pulse more regular, rather quick, with slight degree of fever, occasional expectoration of blood, with small pieces of apparently a solid membranous substance. Complained of great soreness of mouth and throat: ordered an enema, with an emollient gargle for the mouth and throat, and to increase the dose of magnesia.

20th.—Vomiting only occasionally; pain considerably abated; had a natural evacuation per anum; fever less; pulse regular, principally complaining of the mouth and throat; kept some gruel down; had procured rest during the night: continued the magnesia in larger doses.

21st.—I found her up, and quite comfortable: continued strictly the antiphlogistic regimen. She gradually gained strength, and is now perfectly recovered, with the exception of occasional spasm of the stomach and slight soreness of the mouth and throat.

*Remarks.*—On strict enquiry, I found she had taken at first three ounces of the sulp. acid. dil.; but, finding that not sufficiently efficacious to accomplish her design, she obtained one ounce of the sulp. acid. fort., but could not get down more than about three parts of it, from the irritation produced on the fauces, &c.

I do not submit the present case from any novelty in the practice adopted, but from the circumstance of her so speedily recovering of those violent symptoms produced on the immediate taking of so great a quantity of acid, and the action of the stomach not being now in any manner impaired from performing its due functions after so considerable a derangement.

I should mention, that the patient was a person addicted to the use of spirituous liquors; and, perhaps, the coats of the stomach may have been in some measure indurated by the too free use of liquor of the most deleterious kind.

*Plymouth Dock; April* 7, 1818.

